# Understanding the Links between Inferring Mental States, Empathy, and Burnout in Medical Contexts

**DOI:** 10.3390/healthcare9020158

**Published:** 2021-02-03

**Authors:** Naira Delgado, Helena Bonache, Moisés Betancort, Yurena Morera, Lasana T. Harris

**Affiliations:** 1 Departamento de Psicología Cognitiva, Social y Organizacional, Universidad de La Laguna, 38205 La Laguna, Spain; ndelgado@ull.edu.es (N.D.); ymorera@ull.edu.es (Y.M.); 2 Departamento de Psicología Clínica, Psicobiología y Metodología, Universidad de La Laguna, 38205 La Laguna, Spain; moibemo@ull.edu.es; 3 Instituto de Neurociencia Cognitiva (IUNE), Universidad de La Laguna, 38205 La Laguna, Spain; 4 Experimental Psychology, University College London, London WC1H 0AP, UK; lasana.harris@ucl.ac.uk

**Keywords:** empathy, mental states inferences, burnout, healthcare professionals

## Abstract

It is generally accepted that empathy should be the basis of patient care. However, this ideal may be unrealistic if healthcare professionals suffer adverse effects when engaging in empathy. The aim of this study is to explore the effect of inferring mental states and different components of empathy (perspective-taking; empathic concern; personal distress) in burnout dimensions (emotional exhaustion; depersonalization; personal accomplishment). A total of 184 healthcare professionals participated in the study (23% male, *M*age = 44.60; *SD* = 10.46). We measured participants’ empathy, the inference of mental states of patients, and burnout. Correlation analyses showed that inferring mental states was positively associated with perspective-taking and with empathic concern, but uncorrelated with personal distress. Furthermore, emotional exhaustion was related to greater levels of personal distress and greater levels of inferences of mental states. Depersonalization was associated with greater levels of personal distress and lower levels of empathic concern. Personal accomplishment was associated with the inference of mental states in patients, lower levels of personal distress, and perspective-taking. These results provide a better understanding of how different components of empathy and mental state inferences may preserve or promote healthcare professionals’ burnout.

## 1. Introduction

The COVID-19 pandemic has brought an extraordinary pressure on hospitals, with specialists at the frontline being the most seriously affected. Undoubtedly, the pandemic has accentuated the need to deal with the emotional wellbeing and stress-related problems of healthcare professionals [1]. But even before this critical period, there were unacceptable health problems among healthcare professionals, who are coping with emotionally distressing situations day after day: illness, dying, suffering in every form. This painful reality can lead to compassion fatigue, burnout, and professional distress and result in a low sense of accomplishment and severe emotional exhaustion [2,3,4]. 

Previous literature has explored the impact of empathy in emotional exhaustion [5], establishing links between empathy and burnout [6,7]. However, empirical evidence has shown both negative and positive links between high burnout scores and empathy [8,9]. These inconsistencies make it quite difficult to establish the direction and nature of the relationship between empathy and burnout [10]. The main objective of this research is to clarify the effect of different empathic components and the inference of patients’ mental states in healthcare professionals and their link with burnout dimensions. Specifically, we propose that the relationship between empathy and burnout depends on whether the dimensions of empathy are self-oriented (personal distress) or other-oriented (perspective-taking and empathic concern). The general hypothesis is that the self-oriented components of empathy will be positively related to burnout dimensions, whereas the other-oriented components will be negatively associated with them.

Empathy is considered a multidimensional construct, though there is no single classification of its dimensions. Davis [11] developed an individual difference measure of empathy (the Interpersonal Reactivity Index, IRI) based on the idea that empathy can be considered as a set of related constructs. Perspective-taking is the tendency to adopt others’ point of view, or knowing another person’s internal states. Fantasy is the ability to transpose oneself imaginatively into the feelings and actions of fictitious characters. Empathic concern is the tendency to experience feelings of warmth, compassion, and concern for other people, assessing the other-oriented approach of empathy, whereas personal distress refers to a self-oriented approach to empathy, including feelings of distress and unease when observing others’ suffering. Interestingly, all of these constructs imply a reaction towards others, but they are also specific and distinguishable from each other, capturing different components of our relational orientation towards others related to empathy. In the same line, other researchers have established the multidimensionality of empathy [12,13,14,15,16].

Research on social cognition has indicated that empathy and theory of mind processes rely on networks associated with inferring mental states of others [17]. The inference of mental states refers to the assumption that others with whom we interact have minds, which means that they have intentions, plans, and goals [18,19], as well as experiences of pleasure, pain, and emotions [20]. Such inferences occur without cognitive effort and may serve to distinguish people from all other agents and to predict their actions toward us [21,22]. Despite possessing an impressive, unique capacity to reason about the minds of others [23,24,25], people may routinely fail to use this capacity by denying others’ mental states [26,27,28,29,30,31]. This tendency not to perceive minds in others can be conceived as a form of passive dehumanization [25]. The scarce literature that analyzes the relationship between empathy and the inference of mental states has indicated that the cognitive component of empathy (particularly other-focused empathy) is linked to mental state inferences, while the emotional component of empathy (self-focused affect) is unrelated to inferring mental states [32,33]. Previous literature has shown that the role of mental state inferences and empathy is very controversial in medical performance. On the one hand, some studies have shown that being empathic and inferring mental states of patients has a positive impact upon healthcare professionals, who tend to provide better care, experience less distress, and suffer burnout to a lesser extent [34,35,36]. On the other hand, other studies suggest that healthcare professionals reduce their empathy and the inference of mental states in patients to regulate their personal emotions toward suffering, death, and pain, as well as to improve their performance [37,38]. Therefore, patient dehumanization may attenuate the stress and burnout derived from perceiving patients’ physical and psychological pain [39]. In this same vein, it has been argued that depersonalization as a dimension of burnout is linked to the objectification and dehumanization of patients, as a defense and protection mechanism, altering empathy [40]. However, there is a lack of empirical evidence showing whether this mechanism of passive dehumanization is a useful strategy in the medical context or, on the contrary, whether it has severe negative consequences, not only for patients [37] but also for healthcare professionals. 

One of the reasons that may explain these inconsistent results is the breadth of the term empathy. In this sense, those studies that consider empathy as a multidimensional factor have shown that each component of empathy is differently related to the dimensions of burnout. For example, Duarte et al. [41] found that negative self-oriented emotions elicited by others’ distress are associated with burnout and compassion fatigue. Similarly, research with clinical social workers has found that emotional exhaustion is negatively linked to perspective-taking, while personal distress predicts high emotional exhaustion and depersonalization, but low personal accomplishment, while empathic concern is unrelated to any dimension of burnout [42]. Another study also indicated that personal distress increases emotional exhaustion and depersonalization and decreases personal accomplishment in medical students. However, in this case, emotional concern predicts low emotional exhaustion and depersonalization, whereas it predicts high personal accomplishment [43]. As a whole, these results emphasize the need to further analyze the links between self- and other-oriented components of empathy and the dimensions of burnout in medical contexts.

The current study was aimed at addressing the gaps in the literature by analyzing the relationship between different components of empathy, the inference of mental states, and the dimensions of burnout. Specifically, we hypothesized that perspective-taking (other-oriented component) will be negatively and directly related to emotional exhaustion and depersonalization, but positively linked to personal accomplishment (Hypothesis 1). By contrast, the more personal distress participants show (self-oriented component), the more emotional exhaustion and depersonalization they will display, and the less personal accomplishment they will exhibit (Hypothesis 2). In accordance with previous literature, our analysis focuses on the indirect paths from components of empathy to dimensions of burnout through the inference of mental states in patients. In particular, we predicted that participants who exhibit more personal distress (Hypothesis 3a) but less perspective-taking (Hypothesis 3b) will show higher levels of emotional exhaustion, because they infer their patients’ mental states less. On the contrary, those healthcare professionals who exhibit lower levels of personal distress (Hypothesis 4a) but higher levels of perspective-taking (Hypothesis 4b) will display more personal accomplishment, because of the inference of their patients’ mental states. The direct and indirect links between empathic concern and burnout were also examined. However, no specific hypotheses were put forward because of the inconsistency and scarcity of previous research.

## 2. Materials and Methods 

### 2.1. Procedure and Participants

We conducted a cross-sectional, correlational study using an online survey, hosted by Qualtrics (Qualtrics Labs Inc., Provo, UT, USA). An email invitation was sent to five professional nursing and medical associations situated in Spain. The survey link was distributed along with indications that the main purpose of the study was to increase knowledge about how they deal with the task of caring for and supporting their patients. Participants were a convenience sample of healthcare professionals who responded to this self-reported questionnaire; responses were received before (75.7%) and during (24.3%) the first months of the COVID-19 pandemic (to ensure that there were no significant differences between participants due to the response period, ANOVAs were conducted. Results showed that both groups had similar responses in all the variables, except in mental state inferences. This variable showed higher scores for the group that answered the questionnaire during the pandemic than for the group that answered it before the pandemic, F (1174) = 4.79, *p* = 0.03.).

Although 240 questionnaires were received, 56 participants were eliminated because their responses were incomplete. One hundred eighty-four participants fully completed the online survey. Most participants identified as Spanish (97%) and only five individuals indicated being from other countries. The detail of the demographics in this study is shown in Table 1. 

### 2.2. Ethical Approval

Once participants had clicked on the survey link, they received detailed information about the study: the research team, contact details, and general goals. Anonymity of the responses and confidentiality of data were assured, all participants were treated in accordance with APA ethical guidelines, and informed consent was obtained from all volunteers prior to their participation. Moreover, prior to conducting this study, approval was obtained from the Animal Welfare and Research Ethics Committee of the first author’s university.

### 2.3. Instruments

**Empathy.** Participants completed the Spanish adaptation [44] of the Interpersonal Reactivity Index (IRI) [11]. As in previous studies, three of the four dimensions of empathy were selected [3,45], excluding the dimension of fantasy, given that it consists of the tendency of identifying with fictional characters [44]. Moreover, targets of empathy in the original items (e.g., people, someone) were replaced with the word *patients* to assess empathy in healthcare situations. This 21-item instrument comprises three specific aspects of empathy: *Empathic concern* (EC; e.g., “When I see a patient being treated unfairly, I sometimes don’t feel very much pity for them”), *Personal distress* (PD; e.g., “I sometimes feel helpless when I am in the middle of a very emotional situation”), and *Perspective-taking* (PT; e.g., “When I’m upset at a patient, I usually try to “put myself in his shoes” for a while”). The response scale ranged from 1 (*strongly disagree*) to 5 (*strongly agree*). The internal consistency omega hierarchical was adequate for each of its subscales (EC = 0.65; PD = 0.75; PT = 0.76). 

**Burnout.** The Spanish version of Maslach Burnout Inventory (MBI) [46] was used to measure professional burnout. The MBI includes three scales: *Emotional exhaustion* (EE; nine items, e.g., “Feel emotionally drained from work”), *Depersonalization* (D; five items, e.g., “Don’t really care what happens to patients”), and *Personal accomplishment* (PA; eight items, e.g., “Deal with emotional problems calmly”). This 22-item self-report scale assessed the frequency with which respondents experienced burnout-related feelings or emotions on a seven-point scale (0 = *never*; 6 = *daily*). Coefficient omega hierarchical was 0.93 for the entire sample, and 0.90 for EE, 0.73 for D, and 0.83 for PA subscales.

**Mental state inferences.** After consulting empirical research on mental state inferences and previous scales related to the topic, we generated an item pool. Two of the authors, both experts in dehumanization, reviewed these items independently, deleting redundant ones and selecting those that fit with inferring mental states. Then, the items were edited for clarity and content by the second author. The final instrument, the Mental State Inferences Scale (MSIS), was composed of eight items that explained 51% of variance. Participants indicated how frequently (1 = *never*; 5 = *always*), while talking and interacting with patients, they tend to think about different aspects related to their patients’ mental states. Higher scores indicated higher attribution of mental states to patients. Examples of the items are “The patient has projects and plans” or “The patient needs to give meaning to their life”. A confirmatory factor analysis (CFA) of the MSIS shown a clear one-factor structure (χ^2^/*df* = 31.221, root mean square error of approximation [*RMSEA*] = 0.061, comparative fit index [*CFI*] = 0.98), and the loadings of the items on the factor ranged from 0.32 to 0.57. Coefficient omega for the present sample was 0.86. 

**Background information.** Participants reported their sex, age, nationality, occupation, and years of professional experience. 

## 3. Results

### 3.1. Descriptive Statistics and Zero-Order Correlations

Data analyses were conducted using R (R Core Team, 2020) through ULLRToolbox [47]. Means and standard deviations of all study variables, as well as Pearson’s correlations among all study variables, are shown in Table 2. Results showed that those healthcare professionals who reported more perspective-taking tended to display lower levels of depersonalization, but higher levels of personal accomplishment. However, perspective-taking was not significantly related to emotional exhaustion. By contrast, personal distress was positively linked to emotional exhaustion, while it had a negative relationship with personal accomplishment. Moreover, personal distress was unrelated to depersonalization. These results partially support Hypothesis 1 and Hypothesis 2. Our findings also revealed that those participants who inferred mental states to their patients also showed higher levels of perspective-taking, emotional concern, and personal accomplishment. Furthermore, inferring mental states was uncorrelated with personal distress.

### 3.2. Paths from Empathy to Burnout 

To examine the indirect effects from subscales of empathy to dimensions of burnout through the inference of mental states, several multiple mediation analyses were conducted using the Lavaan package in R [48]. Following recommended procedures, maximum likelihood estimator and standard error were calculated using 1000 bootstrapped samples. Indirect effects were considered statistically significant by inspecting *p*-values and if the 95% confidence interval did not include zero [49]. Three mediation models were examined, one for each dimension of burnout as dependent variable. 

Regarding the model examining emotional exhaustion as the dependent variable (see Table A1), a direct path from personal distress to emotional exhaustion was found (β = 0.30, *p* < 0.001, 95% CI = [0.150, 0.454]). However, and contrary to our predictions, the inference of mental states did not mediate this link (see Table 3). Likewise, no direct or indirect effects were found between perspective-taking and emotional exhaustion. Therefore, neither Hypothesis 3a nor Hypothesis 3b was supported. 

**Figure 1:**
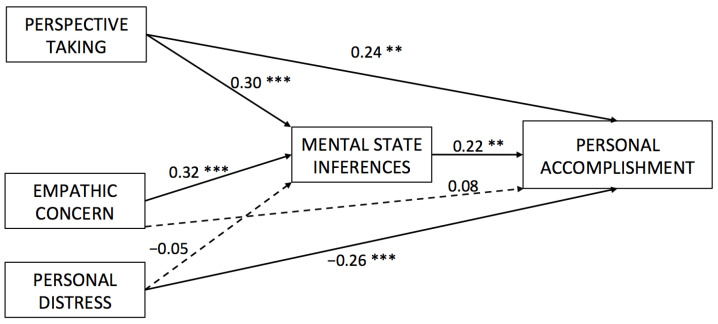
Direct paths from dimensions of empathy to personal accomplishment through the inference of mental states. ** *p* < 0.01, *** *p* < 0.001.

In the model testing mediation from empathy dimensions on personal accomplishment, a negative and direct path was found between personal distress and personal accomplishment (Figure 1; see Table A2). Contrary to Hypothesis 4a, this link was not mediated by the inference of mental states. However, and upholding Hypothesis 4b, we found that healthcare professionals who displayed high levels of perspective-taking and emotional concern also indicated high personal accomplishment when they inferred mental states to their patients (see Table 3). Furthermore, both perspective-taking and empathic concern were positively related to mental states in the three mediation models. Moreover, we examined the relationship between emotional concern and burnout. Neither direct nor indirect effects were found (see Table A1, Table A2 and Table A3). 

Although there were no predictions for depersonalization, it was tested whether the components of empathy were directly and indirectly related to this dimension of burnout (see Table A3). Only perspective-taking was negatively linked to depersonalization (β = −0.22, *p* < 0.05, 95% CI = [−0.399, −0.049]). Moreover, indirect effects through the inference of mental states were not found (see Table 3). 

## 4. Discussion

Empathy in healthcare and the role it plays in the development of personal costs is a complex phenomenon. The purpose of this study was to analyze the relationship between three empathic components, mental state inferences, and burnout dimensions. The general hypothesis was that inferring mental states generates different consequences for professionals’ wellbeing, depending on the type of empathic component that healthcare professionals display.

Our study showed that mental state inferences lead to positive effects for healthcare professionals. These results suggest that being conscious of a patient’s mental state, thoughts, and feelings does not imply personal costs. On the contrary, it increases the likelihood of finding the work significant and gratifying. This result is consistent with theoretical literature that suggests that mentalizing patients is a functional coping strategy with positive consequences for the doctor–patient relationship. In this sense, thinking about patients as agents without mind is not an effective strategy to reduce burnout in healthcare workers [34,50]. 

The results also highlight the importance of studying the effects of different empathic components. Specifically, more personal distress is related to less inference of patients’ mental states, and subsequently to negative consequences for healthcare professionals, increasing their levels of emotional exhaustion. On the contrary, perspective-taking seems to increase mental state inferences of patients, resulting in positive effects such as higher levels of personal accomplishment. Our results indicate that negative consequences of empathy in healthcare are circumscribed to a particular component of empathy: personal distress. This component is an aversive, self-focused emotional reaction unrelated to what the other feels and thinks, since it is the expression of what the self is suffering in a particular situation. This empathic component is generally negatively related to social functioning [51]. An important question that remains unexplored is whether the capacity to be affected by the observable or inferred affective experiences of others is necessary to calibrate an appropriate caring response [52]. These findings are consistent with theoretical approaches that define empathy in patient care as an overriding cognitive attribute that presumes an understanding (rather than feeling) of the patient’s suffering, combined with the ability to communicate this understanding, and the disposition to help [53,54]. From this perspective, personal distress should remain apart from the definition of empathy in healthcare, since it is a distinguishable concept with extremely negative consequences. According to our results, personal distress while helping others has negative consequences for healthcare professionals’ wellbeing. For that reason, it should not be considered in programs or interventions aimed at improving empathic skills. 

One of the novelties of this study is that it incorporates mental state inferences in the relationship between empathy and burnout. The finding that emotional concern and perspective-taking increase personal accomplishment in healthcare professionals when they infer the mental state of patients is remarkable. It suggests that paying attention to the feelings of others while thinking about patients’ minds increases the levels of professional wellbeing and satisfaction at work. Importantly, our results make compatible patients’ needs and healthcare professionals’ needs: empathic relationships reinforce patient involvement and satisfaction with the therapeutic process, improve quality of care, and reduce medical errors [55].

In our view, these results invite us to cautiously reconsider previous conclusions about the relationship between dehumanization and burnout [39,50]. In line with our results, it is important to note that mentalizing the patients is not necessarily the cause of higher levels of burnout. Instead, our findings seem to indicate that perspective-taking, mental state inferences, and empathic concern play a positive role in protecting healthcare workers from burnout. Sadly, healthcare professionals assume that taking personal distance is a necessary response to avoid emotional exhaustion. However, empirical evidence reveals that inferring mental states and perspective-taking could be ways to reduce personal distress, probably because the attentional resources are allocated to the patient rather than to the self. Advances in neurocognitive science are an extraordinary opportunity to improve our knowledge of the different components of empathy and their neural correlates [56]. Further empirical research in this field could help us to understand the role of different components of empathy in specific psychosocial risks at work. Also, future studies should explore other personal costs in healthcare professionals that cause severe problems and that remain underexplored (i.e., turnover intentions, abuse of addictive substances, etc.). Finally, more meta-analyses are required to differentiate the specific role of each of the components of empathy in wellbeing beyond the negative consequences of healthcare.

It is highly important that intervention program design be derived from empirical research in this field and lead to a deep change in the culture of empathy in medicine [57]. In this way, systematic reviews have noted that educational and mindfulness-based stress reduction interventions can be effective in increasing empathic skills [58,59]. Consistent with this research, the results of the present study can help occupational health and risk prevention departments to design efficient empathy-based intervention programs aimed at minimizing the potentially negative consequences of healthcare provision. Specifically, these training and education programs for clinicians and nurses should be oriented to the development of perspective-taking and empathic concern. However, it is necessary to conduct more studies to identify the specific strategies that generate greater changes as well as the relationship between those strategies and personal and organizational variables. Differences across empathic components and their consequences should lead to the development of specific tasks for training perspective-taking, empathic concern, and preventing personal distress. 

The current study has some limitations that should be considered when interpreting the results. First, the sample size has not allowed us to make comparisons by specialty, years of experience, and other organizational dimensions that undoubtedly play a relevant role. Second, the selection of additional instruments and methodologies is necessary to improve the conclusion obtained. Particularly interesting is the inclusion of physiological measures [32]. Third, it is not possible to determine the response rate since the sampling frame is unknown. Furthermore, it is difficult to identify whether the reasons why some healthcare professionals decided not to participate in the study are relevant factors for the purpose of the study. This issue may introduce bias into data interpretation [60]. Finally, this is a correlational study, and to draw causal links between variables goes beyond its purpose.

Although the current study has several limitations, it also provides new research directions. Since the COVID-19 pandemic, society has become more conscious of the imperative of caring for healthcare professionals. The emotional costs of helping need to be reduced, and research on empathy and its consequences plays a relevant role in implementing this goal. Healthcare professionals are constantly trying to deal with their own personal emotions towards suffering, pain, and death, while at the same time trying to provide the best care for their patients. They undoubtedly face an important challenge, and research on this topic could help to clarify and improve our understanding of how they can leverage empathy to achieve positive professional outcomes and at the same time maintain personal wellbeing. 

## 5. Conclusions

This study provides unique empirical evidence of the variables related to burnout in healthcare professionals. It demonstrates how dimensions of empathy are differently related to types of burnout, and that these links may be mediated when inferring mental states to patients. In particular, our results emphasize the positive role that other-oriented empathy plays, and show how the inference of patients’ minds affects healthcare professionals’ appraisal of personal achievement. Training on how to manage empathy to be more focused on others and less focused on the self can lead to more effective healthcare provision, impacting patients and healthcare professionals’ quality of life.

## Figures and Tables

**Table 1:** Demographics of study sample.

	Frequency (*n*)	Percentage (%)	Mean	SD	Range
Age			44.6	10.46	23–65
Gender					
Female	142	77.2			
Male	42	22.8			
Working experience (years)					
Nurses	105	56.8	19.24	11.31	1–41
Doctors	56	30.3	20.80	9.88	1–40
Nursing assistants	13	7.6	13.69	11.31	1–33
Other categories	10	5.4	20.30	9.35	2–33
Current specialty					
Emergency and intensive care	47	25.54			
Surgery and medical specialties	30	16.30			
Primary care	28	15.28			
Outpatient consultation, outpatient specialty clinic, central clinical division	26	14.13			
Pediatrics and gynecology	15	8.15			
Palliative care	14	7.61			
Mental health	10	5.43			
Others	14	7.61			

**Table 2:** Means, standard deviations, and correlations between study variables.

	Mean	SD	MenSta	PerTak	EmoCon	PerDis	EmoExh	Dep	PerAcc
MenSta	3.88	0.66	-						
PerTak	3.73	0.77	0.41 ***	-					
EmoCon	4.00	0.74	0.40 ***	0.30 ***	-				
PerDis	1.99	0.70	−0.13	−0.23 ***	−0.08	-			
EmoExh	3.40	1.68	0.14	0.01	0.11	0.27 ***	-		
Dep	2.46	1.52	−0.14	−0.29 ***	−0.19 **	0.14	0.47 ***	-	
PerAcc	6.14	0.99	0.38 ***	0.42 **	0.27 ***	−0.35 ***	−0.29 ***	−0.41 ***	-

*Note.* MenSta = mental state inference; PerTak = perspective-taking; EmoCon = emotional concern; PerDis = personal distress; EmoExh = emotional exhaustion; Dep = depersonalization; PerAcc = personal accomplishment. ** *p* < 0.01; *** *p* < 0.001.

**Table 3:** Indirect paths from dimensions of empathy to burnout dimensions through the inference of mental states for the models of multiple mediation analyses.

	Estimate	*z*-Value	P (>|z|)	Std	95% CI
Paths for Emotional Exhaustion					
PerTak→InfMen→EmoExh	0.047	1.334	0.166	0.036	[−0.022, 0.117]
EmoCon→InfMen→EmoExh	0.045	1.403	0.138	0.032	[−0.018, 0.108]
PerDis→InfMen→EmoExh	−0.006	−0.459	0.653	0.012	[−0.029, 0.018]
Paths for Personal Accomplishment					
PerTak→InfMen→PerAcc	0.067	2.221	0.026	0.030	[0.008, 0.127]
EmoCon→InfMen→PerAcc	0.072	2.338	0.019	0.031	[0.012, 0.132]
PerDis→InfMen→PerAcc	−0.010	−0.574	0.566	0.018	[−0.045, 0.024]
Paths for Depersonalization					
PerTak→InfMen→Dep	0.007	0.208	0.835	0.032	[−0.056, 0.070]
EmoCon→InfMen→Dep	0.007	0.209	0.835	0.032	[−0.056, 0.069]
PerDis→InfMen→Dep	−0.001	−0.196	0.845	0.005	[−0.010, 0.008]

*Note*. EmoCon = emotional concern; PerTak = perspective-taking; PerDis = personal distress; EmoExh = emotional exhaustion; PerAcc = personal accomplishment; Dep = depersonalization; InfMen = inference of mental states.

## Data Availability

The data presented in this study are available on request from the corresponding author.

## Appendix A

**Table A1:** Multiple mediation analyses model for direct effects from dimensions of empathy to emotional exhaustion through the inference of mental states.

Paths	Estimate	*z*-Value	P (>|z|)	Std	95% CI
EmoCon→EmoExh	0.085	0.991	0.991	0.085	[−0.083, 0.252]
PerTak→EmoExh	0.085	−0.397	0.692	−0.034	[−0.201, 0.134]
PerDis→EmoExh	0.077	3.905	0.000	0.302	[0.150, 0.454]
EmoCon→InfMen	0.072	4.292	0.000	0.307	[0.167, 0.447]
PerTak→InfMen	0.074	4.371	0.000	0.323	[0.178, 0.467]
PerDis→InfMen	0.079	−0.478	0.633	−0.038	[−0.192, 0.116]
EmoExh→InfMen	0.094	1.565	0.118	0.147	[−0.037, 0.331]

*Note*. EmoCon = emotional concern; PerTak = perspective-taking; PerDis = personal distress; EmoExh = emotional exhaustion; InfMen = inference of mental states.

**Table A2:** Multiple mediation analyses for direct effects from dimensions of empathy to personal accomplishment through the inference of mental states.

Paths	Estimate	*z*-Value	P (>|z|)	Std	95% CI
EmoCon→PerAcc	0.070	1.195	0.232	0.084	[−0.054, 0.022]
PerTak→PerAcc	0.069	3.442	0.001	0.237	[0.102, 0.372]
PerDis→PerAcc	0.071	−3.710	0.000	−0.263	[−0.401, −0.124]
EmoCon→InfMen	0.070	4.563	0.000	0.322	[0.183, 0.460]
PerTak→InfMen	0.077	3.917	0.000	0.302	[0.151, 0.453]
PerDis→InfMen	0.073	−0.626	0.531	−0.045	[−0.188, 0.097]
PerAcc→InfMen	0.085	2.616	0.009	0.223	[0.056, 0.390]

*Note*. EmoCon = emotional concern; PerTak = perspective-taking; PerDis = personal distress; EmoExh = emotional exhaustion; InfMen = inference of mental states.

**Table A3:** Multiple mediation analyses for direct effect from dimensions of empathy to depersonalization through the inference of mental states.

Paths	Estimate	*z*-Value	P (>|z|)	Std	95% CI
EmoCon→Dep	0.102	−1.451	0.147	−0.148	[−0.347, 0.052]
PerTak→Dep	0.089	−2.510	0.012	−0.224	[−0.399, −0.049]
PerDis→Dep	0.078	1.049	0.294	0.082	[−0.071, 0.234]
EmoCon→InfMen	0.067	4.727	0.000	0.315	[0.185, 0.446]
PerTak→InfMen	0.073	4.334	0.000	0.317	[0.173, 0.460]
PerDis→InfMen	0.075	−0.575	0.565	−0.043	[−0.191, 0.105]
Dep→InfMen	0.101	0.209	0.834	0.021	[−0.177, 0.220]

*Note*. EmoCon = emotional concern; PerTak = perspective-taking; PerDis = personal distress; EmoExh = emotional exhaustion; InfMen = inference of mental states.
